# Metabolic pathways to sustainability: review of purple non-sulfur bacteria potential in agri-food waste valorization

**DOI:** 10.3389/fbioe.2025.1529032

**Published:** 2025-02-24

**Authors:** Guillaume Bayon-Vicente, Laura Toubeau, Manon Gilson, Guillaume Gégo, Nishitha Landgey, Simone Krings, Baptiste Leroy

**Affiliations:** Laboratory of Proteomics and Microbiology, Research Institute for Biosciences, University of Mons, Mons, Belgium

**Keywords:** purple non-sulfur bacteria, agri-food waste, upcycling, metabolism, carbohydrates, volatile fatty acids, alcohol, biorefineries

## Abstract

Agri-food waste (AFW) represents a significant fraction of the material generated by the agri-food industry, which itself accounts for almost one-third of the annual global anthropogenic greenhouse gas (GHG) emissions. Considering the growing global population and the consequent rise in food demand, the management and valorization of this waste are essential to ensure the sustainability of the entire food chain for future generations. Recycling agri-food waste offers a promising strategy to mitigate the sector’s environmental impact, particularly when the waste consists of food-grade materials that enhance its intrinsic value. Retaining such products within the agri-food chain by converting them into feed or food, a process referred to as “waste upcycling,” is therefore of critical importance. Purple non-sulfur bacteria (PNSB) are emerging as promising candidates for AFW upcycling due to their remarkable metabolic versatility, which allows them to metabolize a wide range of organic substrates, including carbohydrates, volatile fatty acids (VFAs), and alcohols, into valuable microbial biomass. This biomass is notably rich in superior quality proteins, vitamins, pigments, and other high-value compounds. The phototrophic metabolism of PNSB is particularly advantageous for organic matter valorization, as the carbon conversion yield approaches unity by utilizing light as an energy source. This review explores the potential of PNSB in upcycling AFW streams derived from various sources, such as fruit and vegetable residues, as well as effluents from the dairy, brewery, and sugar industries. The pre-treatment methods required to optimize substrate availability are also discussed. Furthermore, we examine the metabolic pathways utilized by PNSB under phototrophic conditions to assimilate the most common carbon substrates found in AFW, highlighting critical gaps in our understanding of their metabolism. Additionally, challenges and opportunities in AFW valorization, with a focus on PNSB applications, are identified. This review underscores recent advancements and ongoing challenges, emphasizing the potential role of PNSB in driving sustainable circular bioeconomy applications for AFW.

## Graphical Abstract

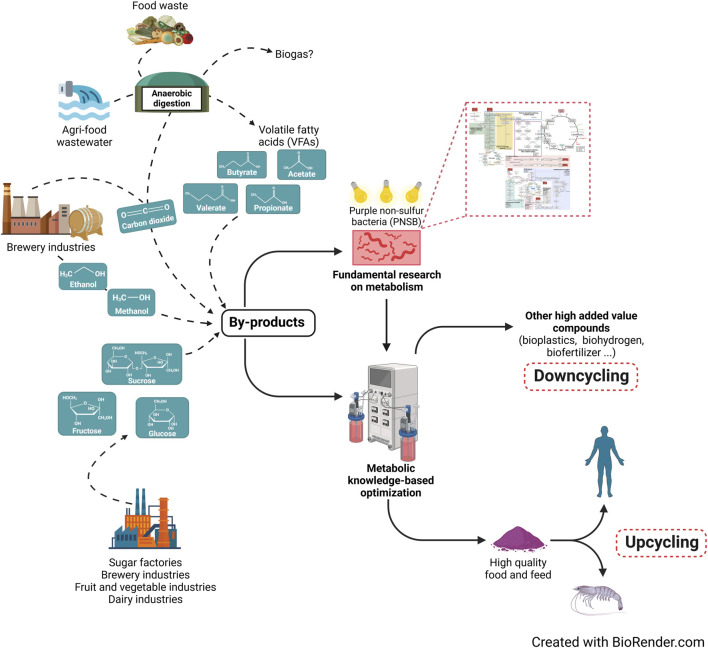

## 1 Introduction

Over the last few decades, waste production has increased dramatically worldwide. The current annual generation of municipal solid waste (MSW) exceeds two billion metric tons, and projections suggest that this amount will continue to increase by around 70% by 2050. This rise in waste production poses significant challenges for waste management systems globally (Alves, 2024).

In 2013, FAO (Food and Agriculture Organization) estimated that one-third of all food produced for human consumption each year, corresponding to 1.3 billion tonnes, is either lost or wasted throughout the entire food supply chain. This includes organic waste generated during production, post-harvest handling and processing, storage, transport, distribution, household processing, and consumption (Ravindran and Jaiswal, 2016; Stenmarck et al., 2016). Despite its large-scale production, a significant amount of biowaste is not recycled (Bank the world, 2024). Agri-food waste (AFW) primarily originates from plant sources, including peels, leaves, seeds, pomace, and from animal products such as meat derivatives, feather or egg products (Berenguer et al., 2023) to a lesser extent. The fruit and vegetable sectors are significant contributors to food waste, as an estimated 45% of products are lost during production and/or as leftovers (Bank the world, 2024). Aside from representing a substantial economic loss estimated at $750 billion per year (excluding fish and seafood) (FAO. Food Wastage Footprint, 2013), the treatment and disposal of AFW presents a significant environmental impacts by wasting resources such as land and water, or by increasing greenhouse gas emissions (GHG). Food losses in industrialized countries are surprisingly similar to those in developing countries. However, in developing countries, over 40% of these losses take place during post-harvest and processing stages, whereas in industrialized countries, over 40% occur at the retail and consumer stages (Gustavsson et al., 2011). In Europe, households are the largest contributors, responsible for 54% of total food waste, followed by industrial processing at 19%. The remaining 27% are distributed among food service (11%), production (8%), and wholesale and retail sectors (8%) (Stenmarck et al., 2016). These alarming statistics highlight the urgent need for alternative waste management solutions. As a sign of the importance given to AFW management, two major institutions (i.e., the European Commission and the United Nations Environment Program) emphasize the critical importance of the development of efficient AFW management practices. Indeed, while the European Commission has prioritized food waste in its Action Plan for the Circular Economy Strategy (European commission, 2018), the United Nations 2021 report highlights that using these by-products can enhance the nutritional value of new food formulations, help reach sustainable development goals by reducing waste and GHG emissions, and improve global food supply security (UNEP United Nations Environment Programme, 2021).

Nowadays, 40%–50% of AFW are composted or landfilled or could be valorized as animal feed (Truong et al., 2019) as well as for the production of biofuels (biogas, biohydrogen, bioethanol, biodiesel) and commodity chemicals (Dahiya et al., 2018). Solid waste might also require pre-treatment steps (Ng et al., 2020; Areniello et al., 2023) such as dark fermentation or anaerobic digestion (Weide et al., 2019), or physicochemical, including ultrasounds (Li et al., 2013) or hydrothermal treatment (Sánchez et al., 2023). Even if energy-intensive, incineration is also performed (Commission of the European Communities, 2008). Some AFW are also attracting attention as source of bioactive molecules for the cosmetic and pharmaceutical sectors as sustainable and natural source of pigments, aromas, and antioxidants (Saini et al., 2019; Genkinger et al., 2004) but this only utilizes a small proportion of the available feedstocks.

Particularly within the circular economy framework, the concept of “upcycling” is gaining significant attention. For food-grade by-products or waste, any recycling process that results in valorization outside the food chain (e.g., for bioenergy, bioplastics, or fertilizers) should be considered a downcycling process. Upcycling AFW—by converting them into new food or feed products, ideally of higher nutritional quality—offers substantial potential to reduce the environmental and economic impacts of the agri-food sector. This is achieved both directly, by mitigating the waste disposal impact mentioned earlier, and indirectly, by reducing the need for additional agricultural production (Tsegaye et al., 2021). In this context, identifying effective strategies to upcycle AFW through microbial conversion has become a significant focus area, as it aligns with the principles of a circular bioeconomy (Ng et al., 2020; Areniello et al., 2023). Over the last few decades, purple non-sulfur bacteria (PNSB) have garnered significant interest in biotechnology due to their remarkable metabolic versatility, making them highly promising for several applications [reviewed in Capson-Tojo et al. (Capson-Tojo et al., 2020)] including wastewater treatment, fertilizer production, bioplastic synthesis, hydrogen generation, and even environmental control and life support system (ECLSS) for the MELiSSA project lead by the European Space Agency (ESA) (Hendrickx et al., 2006). Considering the superior quality of PNSB protein, those organisms are also of major interest for potential food application (Hülsen et al., 2022). Indeed, PNSB biomass has recently been shown to exhibit an amino acid profile matching human diet and particularly in term of essential amino acids (dietary match >100 for most of the essential amino acid, essential amino acid index >1) (Spanoghe et al., 2021).

PNSB represent a heterogeneous group of Gram-negative proteobacteria capable of sustaining various metabolic modes, such as photo-organo-heterotrophy, photo-litho-autotrophy, dark fermentation, and (an)aerobic respiration (Koblížek, 2009; Cerruti et al., 2023). Depending on environmental and physiological conditions, PNSB can thrive in either oxic or anoxic environments, utilizing a wide array of electron donors, ranging from organic to inorganic compounds. Furthermore, energy production can be achieved using either light (phototrophy) or chemical compounds (chemotrophy). Optimal growth is typically observed under photo-organo-heterotrophy (hereafter referred to as photoheterotrophy), where organic molecules serve as both electron and carbon sources. Phototrophic growth offers a major advantage for upcycling organic matrix like AFW. Indeed, PNSB exhibit near-perfect substrate-to-biomass COD conversion when grown photoheterotrophically. For instance, a comparison of biomass production yields between chemoheterotrophy and photoheterotrophy strongly favor the latter, with yields of 0.6 g COD_biomass_ per g COD_nutrient_ and 0.9–1.1 g COD_biomass_ per g COD_nutrient_, respectively (Capson-Tojo et al., 2020; Hülsen et al., 2022; Alloul et al., 2019). Ratio higher than 1 in term of biomass COD conversion is explained by the dependency of purple bacteria to bicarbonate ions and thus its assimilation in the presence of reduced carbon source (De Meur et al., 2020; Bayon-Vicente et al., 2020a). This efficiency highlights their significant potential for converting AFW into high-value biomass for food or feed application.

The scope of this review is to evaluate and discuss the potential of PNSB for the valorization of some AFW. In particular, we aim to bridge the gap between bioengineering and fundamental science linking composition of the selected AFW with metabolic capabilities of PNSB. Through this non-exhaustive review, our goal is to identify either which AFW are the most promising for PNSB valorization because metabolic capabilities are already well known and understood, or which metabolic pathway should be further explored because the lack of fundamental understanding today impairs the valorization of abundant resources. As our goal was to link PNSB metabolic capabilities with the valorization of AFW and not to offer an exhaustive list of these waste stream [already reviewed before (Sarker et al., 2023)], a selection was made regarding discussed AFW which was based on different criteria: i) upcycling in food/feed product was possible, ii) they are currently poorly valorized, iii) they contain potential carbon source for PNSB and iv) they are mentioned as abundant waste stream by either the FAO 2011 report on “Global food losses and food waste” (Gustavsson et al., 2011) or by the European Eurostat database (Stenmarck et al., 2016). To present the metabolic knowledge or gaps in metabolic capabilities linked with valorization of these AFW, we focus on the photoheterotrophic metabolism (the most relevant for AFW upcycling) of the most extensively studied PNSB, namely *Rhodospirillum rubrum* (*Rs. rubrum*), *Rhodobacter capsulatus* (*Rh. capsulatus*), *Cereibacter sphaeroides* (*Ce. sphaeroides*, formerly *Rhodobacter sphaeroides*), and *Rhodopseudomonas palustris* (*Rp. palustris*).

## 2 Carbohydrate metabolism in purple non-sulfur bacteria, opportunities and challenges for agri-food by-product upcycling

### 2.1 Carbohydrate metabolism in purple non-sulfur bacteria

**FIGURE 1:**
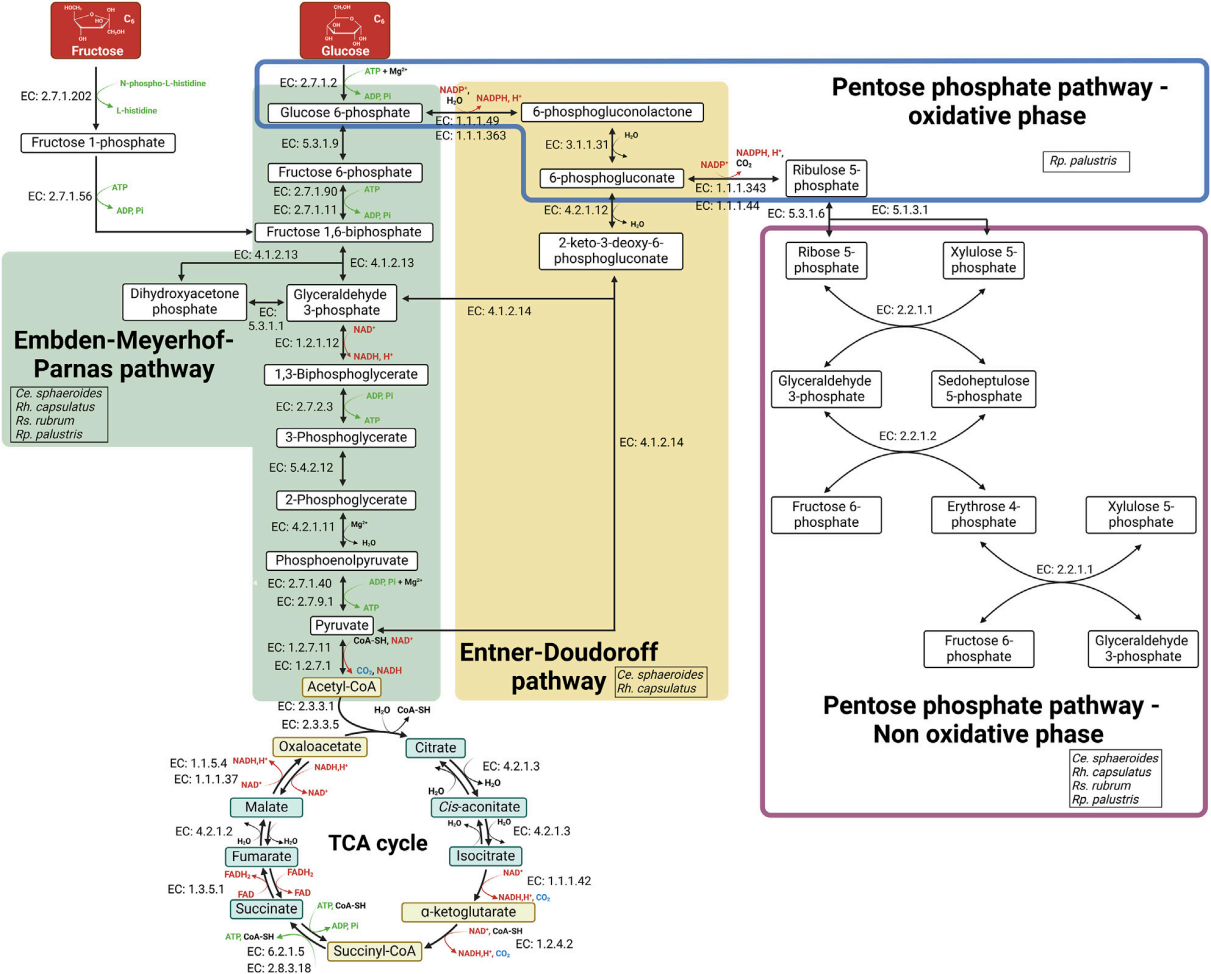
Schematic representation of the metabolic pathways used by PNSB for monosaccharide assimilation. The Embden-Meyerhof-Parnas (EMP) and Entner-Doudoroff (ED) pathways are shown in green and yellow respectively. The Pentose phosphate pathway oxidative and non-oxidative phases are circled on blue and red respectively. The enzymes catalyzing each reaction are indicated by their Enzyme Commission (EC) number. Abbreviations: CoA-SH, coenzyme A; Pi, orthophosphate. Created in https://BioRender.com.

PNSB species assimilate sugars in different ways which also depend on environmental conditions. Metabolic pathways used by bacteria to assimilate sugars are the Emden-Meyerhof-Parnas (EMP) pathway (also called glycolysis), the Entner-Doudoroff (ED) pathway, and the pentose phosphate pathway (PPP) (Figure 1). Understanding the assimilation of monosaccharides by PNSB is a key element for improving the upcycling of sugar-rich biowaste. In addition, given that biowaste often contain sugars as disaccharides or polysaccharides, the capacity of PNSB to assimilate them directly, or after a pre-treatment step to release monosaccharides, is also important (Tulun and Bilgin, 2018; Hamley-Bennett et al., 2016; Guan et al., 2018). It is important to mention here that sugar upcycling, thus targeting the highest substrate-to-biomass conversion rate, is foreseen under phototrophic conditions in PNSB where sugar thus mainly serve as a carbon/electron source and are not oxidized for ATP production.

The two most common monosaccharides found in biowaste are glucose and fructose, which can be present as monosaccharides or disaccharides, such as sucrose in co-products from the sugar industries (see section below). Fructose is assimilated very efficiently by PNSB. *Rs*. *rubrum* (Saier et al., 1971)*, Rh. capsulatus* (Conrad and Schlegel, 1977a) and *Ce. sphaeroides* (Yang et al., 2021) are all capable of assimilating fructose. In these three species, fructose is transported in the same way via the fructose specific-phosphotransferase system (PTS^Fru^), which phosphorylates fructose into fructose-1-phosphate during its transport. Despite the absence of genes encoding the PTS^Fru^ transport system (Larimer et al., 2004), studies have shown that *Rp. palustris* can grow on fructose (Pintucci et al., 2015; Tian et al., 2010). Given the lack of genes associated with the ED pathway in *Rp. palustris*, fructose is likely assimilated via the EMP pathway, for which it possesses all the necessary enzymes (Larimer et al., 2004). Under phototrophic conditions, fructose is assimilated by the Embden-Meyerhof-Parnas (EMP) pathway in *Rh. capsulatus* (Conrad and Schlegel, 1977a) and *Rs. rubrum* (Gibson and Wang, 1968). In *Ce. sphaeroides*, the metabolic pathways involved in fructose assimilation vary depending on environmental conditions with the EMP pathway being used in photoheterotrophy, while the ED pathway is used under aerobic conditions (Conrad and Schlegel, 1977b) (Figure 1).

While glucose is found in a wider range of carbon sources than fructose, its assimilation by PNSB is far less straightforward than fructose and still controversial in some species. For the transport of glucose, in contrast to that of fructose, no glucose-specific transport system has been detected in PNSB. In the 1970s, a glucokinase activity was detected in *Rh. capsulatus* growing in photoheterotrophy with glucose as carbon source (Conrad and Schlegel, 1977a). This suggests that glucose is likely mediated by a glucose non-specific non-PTS transporter system in *Rh. capsulatus*, which implies the presence of a functional kinase to phosphorylate glucose once it enters the cell. However, a recent study suggests two potential glucose transporters in *Ce. sphaeroides*: a non-PTS transport system that would involve glucokinase but also the PTS^Fru^ transport system. However, the PTS^Fru^ transport system was only involved in the first few hours of the bacterial growth (∼12–24 h). On the other hand, a mutation in the glucokinase coding gene had a greater growth inhibitory effect throughout all the bacterial growth (Yang et al., 2021). Glucose is mainly assimilated via the ED pathway in *Rh. capsulatus* (Conrad and Schlegel, 1974) and *Ce. sphaeroides* (Conrad and Schlegel, 1977b) (Figure 1).

The ability of *Rp. palustris* to assimilate glucose is highly controversial in the literature. Some studies report that *Rh. palustris* cannot assimilate fructose and/or glucose (Wu et al., 2012), which could be attributed to the absence of genes encoding sugar specific transporters and/or hexokinase in its genome, which have not been identified so far (Larimer et al., 2004). In contrast, other investigations demonstrate that it can assimilate these sugars, as shown in *Rp. palustris* strain CQK 01 (Wang et al., 2010a), *Rp. palustris* strain 6A (Pintucci et al., 2015) and in a newly isolated strain, *Rp. palustris* strain P4 (Oh et al., 2002). This suggests that the capacity for sugar assimilation in *Rp. palustris* is strain dependent. However, the way in which glucose is phosphorylated is unknown. As for fructose assimilation, due to the lack of enzymes involved in the ED pathway in the *Rp. palustris* genome, glucose is likely assimilated by the EMP pathway (Figure 1).

The enzymes of the complete pentose phosphate pathway, including the oxidative and non-oxidative phases, were shown to be all present only in *Rp. palustris*. It could therefore represent another sugar assimilation pathway in this species, although it has never been clearly shown that it is involved in the assimilation of glucose, fructose or any other type of sugar (Larimer et al., 2004).

Concerning *Rs. rubrum*, it was initially hypothesized that its inability to assimilate glucose was due to a lack of the enzymes necessary for its metabolism (Gibson and Wang, 1968). However, it was later demonstrated that *Rs. rubrum* does possess a functional glucokinase (Delvalle and Asensio, 1978), raising questions about the reason for this glucose assimilation inability. It has also been demonstrated that *Rs. rubrum* lacks glucose 6-phosphate dehydrogenase (EC. 1.1.1.49/EC. 1.1.1.363), which renders the ED pathway and the oxidative pentose phosphate pathway ineffective for sugar assimilation (Hädicke et al., 2011; Grammel et al., 2003) (Figure 1).

Besides fructose and glucose, other monosaccharides assimilable by PNSB are also present in AFW. Among these monosaccharides, galactose, xylose, and arabinose are commonly present in by-products from brewing as well as fruit and vegetable processing. The growth of *Ce. sphaeroides* on galactose has already been reported (Garimella et al., 2019), as well as for the Rp*. palustris* strain P4 (Oh et al., 2002). Similarly, it has been demonstrated in several studies that *Ce. sphaeroides* (Hu et al., 2021; Pattanamanee et al., 2012; Laocharoen and Reungsang, 2014) and *Rh. capsulatus* (Madigan and Gest, 1978) are capable to assimilate xylose. For arabinose, assimilation has been observed in various strains of *Ce. sphaeroides* (Hu et al., 2021; Laocharoen and Reungsang, 2014). Although these studies demonstrate the ability of certain PNSB species to assimilate a wider range of monosaccharides, the metabolic processes involved in their assimilation have not been thoroughly investigated. In most cases, the research has mainly focused on biohydrogen production yields, rather than exploring the mechanisms of sugar metabolism. Consequently, a detailed understanding of how these alternative sugars are transported into the cell and subsequently assimilated is still lacking. Further research is needed to elucidate the pathways involved in the assimilation of these monosaccharides, which could enhance the efficiency of biowaste upcycling processes (Figure 1).

### 2.2 Carbohydrate-rich agri-food by-products

#### 2.2.1 The sugar industry

Sugar is widely consumed globally as a food sweetener and, due to its irreplaceable role in the global food market, is one of the most important commodities in the food and beverage industries. According to FAO reports from 2023, sugar production reached 180 million tonnes in 2023. The highest sugar producers are Brazil, India, and the European Union, with around 44, 32 and 15 million tonnes of sugar produced annually, respectively (FAO. Statisical Yearbook, 2023).

During the sucrose extraction process from either sugar beet or sugarcane, various by-products are generated. Molasses, a non-crystallized syrup, is one such abundant by-product of the sugar industry, with an estimated 40 kg of molasses produced per tonne of raw material (e.g., sugarcane or sugar beet roots) (Geremew Kassa et al., 2024). Worldwide, the production of molasses from sugarcane and sugar beet reaches nearly 64 million tonnes (Geremew Kassa et al., 2024). As a food-grade by-product, molasses offers several valorization opportunities. Sugarcane molasses can be used as an ingredient in traditional baking, as a fermentation feedstock for the production of alco-chemicals (Castañeda-Ayarza and Cortez, 2017), or as an animal feed additive (Mordenti et al., 2021). Today, up to 90% of sugarcane molasses produced worldwide (Iwuozor et al., 2022; Jamir et al., 2021) and 70% of sugar beet molasses produced in the European Union (Anuja Rameshchand et al., 2022) is used for biofuel production as bioethanol, which represent a downcycling of this valuable by-product.

Molasses is primarily composed of sucrose, but its composition varies significantly depending on the raw material and the processing method (Palmonari et al., 2020). The sugar fraction in sugarcane and sugar beet molasses reach up to around 70.0% of the total dry matter. Sucrose accounts for about 95% of the sugar content in sugar beet molasses but only 78% in sugarcane molasses, indicating a higher proportion of inverted sugar in the latter. Notably, sugar beet molasses contains a higher crude protein content (10.7%–15.6% of total dry weight) compared to sugarcane molasses (2.22%–9.31% of total dry weight) (Palmonari et al., 2020).

Molasses has primarily been investigated for biohydrogen production in the context of valorization by PNSB. Canpolat and Ozturk studied the impact of molasses dilution (e.g., 1, 2, 5, 10, 20 g/L), demonstrating that *Rhodoplanes piscinae* 51ATA can grow in a molasses-containing medium, with hydrogen production exceeding that observed in the presence of glucose or acetate. Similarly, it was shown that *Rhodobacter sphaeroides* O.U.001 could grow in a culture medium containing molasses concentrations of 3, 7, 14, 21, or 28 g/L. Both studies found that higher hydrogen production was associated with higher molasses concentrations but also highlighted that pH control remains a significant challenge as several studies have reported a drop in pH during PNSB growth on molasses. Although these studies have advanced the understanding of molasses valorization by PNSB, none have focused on optimizing critical parameters, such as shaking or light penetration, which are key for enhancing molasses upcycling through PNSB biomass production. The high sugar content of molasses underscores its potential for upcycling into PNSB biomass for food or feed applications. Given that the carbon conversion yield of PNSB approaches 100% under phototrophic growth conditions, this strategy represents an efficient upcycling process, keeping molasses within the food chain and minimizing material loss.

#### 2.2.2 The dairy industry

The dairy industry is based on the processing and manufacturing of raw milk into various products such as yogurt, butter or cheese. This industry processes more than 800 million tonnes of milk each year worldwide with Europe accounting for 22% (∼160 million tonnes) of this production (Ashekuzzaman et al., 2019). Processing of this huge amount of milk comes with the production of large amount of wastewater and by-product (Carvalho et al., 2013). These effluents are often highly concentrated in biological oxygen demand (BOD-40 to 8,240 mg L^−1^), chemical oxygen demand (COD – 430 to 18,045 mg L^−1^) and nutrients (nitrogen – 14–830 mg L^−1^, phosphorus – 9–280 mg L^−1^) further indicating that valorization of those effluents is promising. Milk is an interesting source of high-quality proteins (McGregor and Poppitt, 2013; Davoodi et al., 2016; Nongonierma and FitzGerald, 2015), however, milk and subsequent by-products are also a source of sugar in the form of lactose, representing around 35% of total solids in milk.

Milk whey, also called native whey is obtained from the microfiltration (5–10 kDa) of the skim milk to produce a protein-rich diets. The associate permeate contains most of the milk lactose and non-protein nitrogen, is poorly valorized today and could thus constitute a promising substrate for food-grade PNSB production as some of them can assimilate galactose and glucose.

The cheese industry represents the main milk user, accounting for 36% of the total milk production with a global production of 17 million tonnes of cheese each year. However, the cheese manufacturing industry also constitutes a huge by-product producer. Indeed, the cheese whey, issued from milk transformation into cheese, represents around 85%–90% of the initial milk volume accounting for 185 million tonnes each year (Chiara et al., 2013). Within the cheese industry, two types of whey can be obtained. Indeed, following the required fermenting time, the cheese whey (or sweet whey) is obtained and can be further reused for cottage cheese production. The production of the cottage cheese leads to the release of the second cheese whey, also called cottage cheese whey (or acidic whey). Cheese whey is considered as the most important pollutant in dairy wastewaters, not only because of the high organic load, but also for the volume generated. The cheese whey roughly constitutes a milk solution free of casein (milk protein) and fat exhibiting a high salinity (conductivity close to 8 mS.cm^−1^). Cheese whey pH ranges from 5 (acidic whey–mainly issued from cottage cheese) to 7 (sweet whey- mainly issued from cheese) (Carvalho et al., 2013). Interestingly, sweet whey is rich in lactose (70%–80% DW) but also in protein (9%) (Carvalho et al., 2013; Prazeres et al., 2012; Rocha and Guerra, 2020; Koller et al., 2012). The acidic whey is reported to conserve 60% of the dry matter of the original sweet whey it is derived from, and its salinity is three times higher. However, carbohydrate content (almost 100% lactose) accounts for almost 60% of the dry weight (Carvalho et al., 2013). Even if both the cheese and cottage cheese whey will be further treated through ultrafiltration to recover the protein fraction (Tsermoula et al., 2021); the remaining permeate still possess up to 80% of carbohydrate on a dry weight basis further suggesting that this effluent could be valorized through PNSB cultivation.

Considering the significant amount of lactose in different dairy by-product (whey permeate, sweet and acidic whey, etc) and the increasing proportion of lactose-intolerant people in the population, these by-products could be used for PNSB cultivation as an alternative route for lactose valorization. To the authors’ knowledge, no lactase/galactosidase (EC 3.2.1.23) has been reported in PNSB suggesting that an exogenous lactase activity will be necessary to hydrolyze this disaccharide prior to biomass production. Nevertheless, the use of exogenous lactase in a bioindustrial process seems economically feasible and should not restrain the use of PNSB for lactose valorization. The assimilation of galactose still requires further investigation in most of the PNSB.

#### 2.2.3 The fruit and vegetable industry

Worldwide, fruit and vegetable processing globally generates 16% of the total food processed by-products, most of which constitute edible products which are however today landfilled or incinerated (Gustavsson et al., 2011; Banerjee et al., 2017; Sims et al., 2015). Those wastes are rich in carbohydrates further suggesting that following proper treatment, such as hydrothermal pretreatments (Sánchez et al., 2021), they would constitute ideal substrates for PNSB cultivation. Recently, researchers demonstrated that fruit and vegetable wastes can release up to 50 and 10 gr of reducing sugars, respectively, (i.e. mainly glucose, fructose and xylose) per 100 gr of dried matter after hydrothermal treatment (Sánchez et al., 2023). As fruits exhibit a higher reducing sugars content than vegetables, the fruit manufacturing by-products possess the highest potential for PNSB-based valorization. Among fruits, apples constitutes the main produced and consumed fruits in Europe (Centre for the promotion of imports from devleoping countries, 2003; Konopacka et al., 2010; Czernyszewicz, 2016) and FAO estimated that the world apple production reached over 80 million tonnes in 2017 (Lyu et al., 2020). Moreover, Whereas 75% of all apples are consumed fresh, production of apple juice represents the major apple transformation process (Kammerer et al., 2014) leading to the production of pomace that is today mainly used for animal feed or simply discarded (Kammerer et al., 2014; Bhushan et al., 2008). Interestingly, beside the presence of complex carbohydrates (i.e., cellulose, hemicellulose, pectin and lignin), apple pomace is rich in simple sugars (10.8%–15% of the apple pomace dry weight) such as glucose, fructose (arabinose, galactose or xylose accounting for between (Dhillon et al., 2013; Borujeni et al., 2023; Borujeni et al., 2022; Skinner et al., 2018) and making this product a potential substrate for PNSB production.

Another example of potential by-product valorization by PNSB, is the grapes. Grapes are the fourth most cultivated fruits in the world, representing around 70 million tonnes per year. This fruit is mainly used for wine production which is one of the most produced beverages worldwide with a production of 237 million of hectoliter per year in 2023. France is the biggest wine producer with a production of almost 50 hL per year (Barker, 2024). Researchers showed that the production of 1 L of wine yields between 0.3 and 0.5 kg of wine by-products that are today poorly recycled. Indeed, following grapes pressing, the grape pomace is either directly discarded (white wines) or further fermented to extract pigments (red wine) resulting in the production of around 10 million tonnes of grape pomace in 2019 (Nanni et al., 2021). Like other fruit pomaces, grape pomace is rich in carbohydrates, the composition of which depends on the type of grapes and wine, white wine being richer in sugar than red wine (Nanni et al., 2021). The previously proposed strategy (i.e., hydrothermal pre-treatment) can be used to release pomace contained monosaccharides that can be valorized by PNSB cultivation.

#### 2.2.4 The brewery industry

Barley (*Hordeum vulgare L.*) represents one of the most cultivated cereals in the world, accounting for approximately 156 million tonnes of grain harvested in 2020, intended for both human consumption and animal feed (Serna-Díaz et al., 2020). Aside from animal feeding, beer production represent the main transformation process applied to barley (Yu et al., 2018; Ispiryan et al., 2021) In most of the brewery process, around 20 kg of brewer’s spent grains (BSG) is produced for 100 L of beer making. This by-product represents around 85% of all the residues generated during the brewery process and is mainly dedicated to animal feeding (Sganzerla et al., 2021) with a market value of 40€ per tonne (Buffington, 2014). BSG production is estimated around 39 million tonnes per year, where Europe is accounting for 10% of the whole BSG production (Głowacki et al., 2022). Brewers’ spent grain is mainly composed of non-starch polysaccharides such as hemicellulose and cellulose (32%–50% w/w) and protein (19%–30% w/w) (Lynch et al., 2016). Depending on the cereal variety, time of harvesting as well as the malting and mashing regime, the polysaccharide composition can vary but mainly consist in glucose, arabinose and xylose. Thus, it is possible to extract those monosaccharides in order to valorize BSG into PNSB biomass production through enzymatic treatment using Celluclast, Econase, Spezyme, Depol 740 or Alcalase (Lynch et al., 2016) but the higher complexity of the polysaccharides presents in BSG combined with its solid state might represent a challenge. Nevertheless, considering the low value of the BSG on the market (35€/tonne), the valorization of this by-product by PNSB seems promising.

### 2.3 Challenges and opportunities

Sugar-rich by-products represent promising carbon sources for the cultivation of PNSB as they are locally available almost everywhere on the planet, for a cheap price but today still poorly valorized. Moreover, as described above, sugar-rich by-products are often issued from food-grade processes further pushing the use of those by-products for food or feed final applications, targeting an upcycling of these residues. In this context, PNSB represent a unique opportunity to photo-assimilate these carbon sources with a conversion yield close to 100%.

Metabolic pathways used by PNSB to assimilate carbohydrates can vary significantly, not only depending on environmental conditions but also between different strains of the same species. As already mentioned, in *Ce. sphaeroides*, fructose is assimilated through the EMP in phototrophic conditions, while it is assimilated through the ED pathway in aerobic conditions (Conrad and Schlegel, 1977b). Moreover, several studies show that the capacity of *Rp. palustris* to assimilate fructose and glucose differs among the different *Rp. palustris* strains (Pintucci et al., 2015; Tian et al., 2010; Wu et al., 2012). So far, few studies have focused on the metabolic pathways and transport systems used by PNSB to assimilate sugars. Consequently, these metabolic systems are not well described, as it is the case for xylose, arabinose and galactose assimilation. Thus, due to the limited knowledge on bacterial sugar metabolism and its fluctuation depending on the bacterial species and environmental conditions, predicting bacterial growth remains challenging, especially in the context of developing an industrial process.

When multiple carbon sources are present in a medium, a phenomenon known as catabolite repression (Görke and Stülke, 2008) may occur. This leads to the sequential assimilation of carbon sources, where the presence of a preferentially assimilated carbon source triggers the repression of genes involved in the utilization of the secondly assimilated carbon source. This phenomenon is highly widespread in bacteria and has been notably well described for glucose in *E. Coli* [reviewed in Görke and Stülke (2008)]. This phenomenon has already been reported in PNSB, particularly in *Ce. sphaeroides* S10, in the context of biohydrogen production from food waste containing a mixture of organic molecules (mainly xylose, glucose, and acetic acid). In this study, researchers observed that glucose was preferentially assimilated over xylose and acetic acid. Additionally, glucose was efficiently converted to biohydrogen, while xylose and acetic acid were mainly used for biomass production (Pattanamanee et al., 2012; Ghosh et al., 2017). The complexity of the carbon sources in AFW may represent challenges in predicting bacterial metabolism which has been essentially studied with pure or binary mixtures of carbon sources so far (Conrad and Schlegel, 1977b).

Another challenge working with sugars is the high risk of contamination by environmental mesophilic bacteria. If considering non-food/feed final applications, this contamination can reduce the total process yield without compromising it. However, if food or feed represent the final application, then the process should remain axenic meaning that major effort should be allocated to the prevention of contamination, leading to an increase in process capital and operational expenditure.

Solid AFW (i.e., fruit pomace, brewer’s spent grain) would require pre-treatment such as thermal hydrolysis, which also comes with associated cost. Sugars released through this pre-treatment or initially present in the by-products (in the case of molasses or whey for example) are mainly disaccharide or oligosaccharides implying that the PNSB should be capable to release monosaccharides. In the case of the sucrose contained in several by-products, it was shown that some PNSB such as *Rb. capsulatus* or *Rp. palustris* are able to invert it into glucose and fructose (Oh et al., 2002; Haselkorn et al., 2001; Conrad and Schlegel, 1978) whereas *Rs. rubrum* is not (Schultz and Weaver, 1982). This implies that the use of *Rs. rubrum* for the valorization of sucrose containing by-products would require their pre-treatment by an invertase. Concerning lactose, as already mentioned, no PNSB has been shown to possess a lactase activity so far. Today, only a few studies have reported the assimilation of lactose by purple non-sulfur bacteria (Garimella et al., 2019; Ferreira et al., 2023) and most of them included the presence of *Enterobacter* to first convert lactose into organic acids (Moreira et al., 2022), or used genetically modified PNSB strain (Castillo-Moreno et al., 2018). *Rp. palustris* P4 was demonstrated as assimilating lactose on its own under fermenting conditions (Oh et al., 2002) but it remains a unique case study.

The use of cheese whey notably related to the salt content and the acidic pH further requires dilution and/or buffering of the cheese whey. Indeed, with the exception of some less well characterized halophilic (Asao et al., 2011) and acidophilic (Imhoff et al., 2005; Imhoff, 2001) strains, PNSB are found in freshwater or sediment characterized by a neutral pH (Imhoff et al., 2005). Thus, further research is needed to study the adaptation of the four model PNSB or the potential application of these acidophilic and or halophilic PNSB such as bacteria of the Chromatiaceae and Ectothiorhodospiraceae families or *Rhodoblastus acidophilus* to cheese whey containing medium.

## 3 Volatile fatty acid metabolism in purple non-sulfur bacteria, opportunities and challenges for agri-food by-product upcycling

### 3.1 Volatile fatty acid metabolism in purple non-sulfur bacteria

Volatile fatty acids (VFAs) are aliphatic monocarboxylic acids containing between one and six carbon atoms. This group includes formic (C_1_), acetic (C_2_), propionic (C_3_), (iso)-butyric (C_4_), (iso)-valeric (C_5_), and hexanoic (C_6_) acids. Although formic acid is technically part of this group, its abundance remains low in VFA producing processes due to its rapid conversion into other compounds, such as methane (CH_4_) and carbon dioxide (CO_2_) (Annamalai et al., 2020). Accordingly, this review will focus on VFAs with carbon chains ranging from C_2_ to C_6_.

**FIGURE 2:**
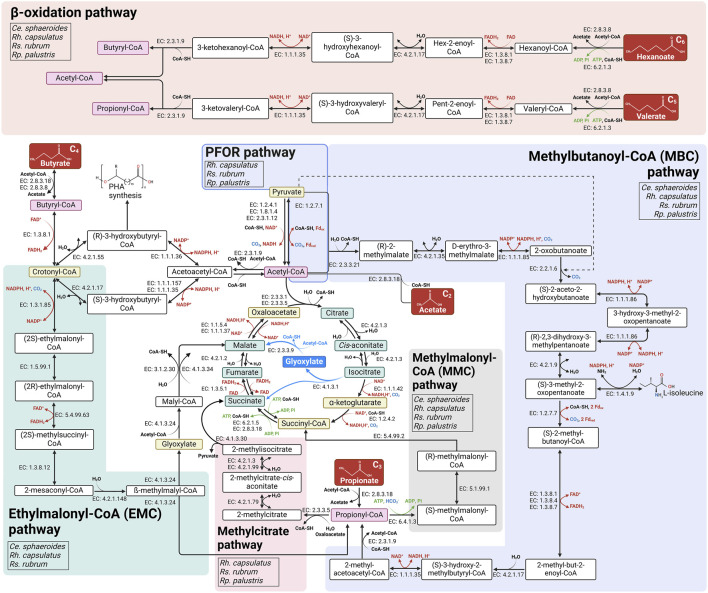
Schematic representation of the metabolic pathways involved in the assimilation of acetate, propionate, butyrate, valerate and hexanoate in four PNSB species. The ethylmalonyl-CoA (EMC), methylbutanoyl-CoA (MBC), methylcitrate, methylmalonyl-CoA, β-oxidation and are shown in green, blue, pink, gray, and orange respectively. The enzymes catalyzing each reaction are indicated by their Enzyme Commission (EC) number. Abbreviations: CoA-SH, coenzyme A; Fdox, oxidized ferredoxin; Fdred, reduced ferredoxin; Pi, orthophosphate. Reactions involving cofactor reduction/oxidation are indicated with red arrows; (de)carboxylation steps are shown in blue, ATP consuming/producing reactions are shown with green arrows. Created in https://BioRender.com.

Acetic acids is usually the mostly abundant VFA produced during fermentation processes. The assimilation of two-carbon compounds such as acetate presents challenges, particularly under photoheterotrophic conditions. Indeed, the TCA cycle involves two decarboxylation steps, catalyzed by isocitrate dehydrogenase (EC: 1.1.1.42) and α-ketoglutarate dehydrogenase (EC: 1.2.4.2) (Figure 2), leading to the inability to achieve net carbon assimilation from acetate. Therefore, anaplerotic pathways are needed when assimilating acetate to bypass these two decarboxylation steps and replenish TCA cycle intermediates used for anabolic processes (Anthony, 2011; Petushkova et al., 2021). Traditionally, four primary anaplerotic reactions have been proposed in PNSB when acetate is the only carbon source: i) the glyoxylate shunt (Kornberg and Krebs, 1957); ii) the ethylmalonyl-Coenzyme A (CoA) pathway (Erb et al., 2007) (EMC pathway); iii) the pyruvate:ferredoxin oxidoreductase (PFOR) (Tang et al., 2011) combined with carboxylating enzymes; and iv) the citramalate cycle (Ivanovsky et al., 1997). The glyoxylate shunt (Figure 2) was the first identified pathway and enables the formation of malate from two acetyl-CoA molecules to replenish the OAA pool (Kornberg and Krebs, 1957). Nevertheless, the expression and presence of the glyoxylate shunt can vary significantly among the strains. Indeed, in *Rp. palustris* (Kornberg and Lascelles, 1960) and *Rh. capsulatus,* ICL is an inducible enzyme that is expressed when the organisms are cultivated on acetate or butyrate. However, in *Ce. sphaeroides* and *Rs. rubrum* (Ivanovsky et al., 1997; Kornberg and Lascelles, 1960; Albers and Gottschalk, 1976) which are isocitrate lyase-negative (*icl* ^−^) bacteria, an alternative metabolic pathway is necessary to enable growth when acetic acid is the carbon source. One of the most-characterized metabolic pathways is the EMC pathway (Erb et al., 2009; Schneider et al., 2012a; Meister et al., 2005; Alber, 2011; Alber et al., 2006) (Figure 2), first described by Erb and collaborators in 2007 (Erb et al., 2007).

The citramalate cycle has long been suggested to be used by *Rs. rubrum* for acetate assimilation (Ivanovsky et al., 1997) but two enzymes necessary for this cycle (i.e., citramalate synthase and mesaconase), have yet to be identified, raising questions about whether the observed early production of citramalate serves a different function (Leroy et al., 2015; Bayon-Vicente et al., 2020b). Recent studies propose that *Rs. rubrum* may instead use the isoleucine biosynthesis pathway, part of the branched-chain amino acid (BCAA) biosynthesis pathway, to assimilate acetate and maintain redox balance during photoheterotrophic metabolism (De Meur et al., 2020; Leroy et al., 2015; Bayon-Vicente et al., 2021; Shimizu et al., 2010; McCully et al., 2019). This more recently discovered pathway aligns with early production of citramalate observed by Berg and Ivanovsky study (Berg and Ivanovsky, 2009) (Figure 2). The PFOR (EC: 1.2.7.1), commonly reported to catalyze oxidative decarboxylation of pyruvate (PA) to generate acetyl-CoA, could represent another acetate assimilation pathway (Leroy et al., 2015) as this enzyme was shown to catalyze the reverse reaction as well, thus producing PA (C_3_) from acetyl-CoA (C_2_) and CO_2_ (Furdui and Ragsdale, 2000; St. Maurice et al., 2007). Leroy et al. reported that in *Rs. rubrum* the PFOR could be involved in the assimilation of acetate producing pyruvate and feeding the BCAA synthesis pathway (Leroy et al., 2015) (Figure 2) or the production of oxaloacetate (OAA) by PA carboxylase (EC: 6.4.1.1) or (indirectly) by PEP carboxylase (EC: 4.1.1.31) or PEP carboxykinase (EC: 4.1.1.32; EC: 4.1.1.49), as shown in *Rh. capsulatus* (Petushkova et al., 2019). Despite the availability of various anaplerotic pathways, only the EMC pathway appeared, through RB-TnSeq, to be essential for acetate assimilation in *Rs. rubrum* (De Meur et al., 2018). Limited information is available on the alternative pathways for acetate assimilation in *Ce. sphaeroides*, however, given its *icl* ^−^ status, it is likely that similar mechanisms to those observed in *Rs. rubrum* are employed. Although *Rh. capsulatus* is recognized as an *icl* ^+^ organism, studies (Petushkova and Tsygankov, 2017; Petushkova and Tsygankov, 2011) suggest it can employ both the glyoxylate cycle and EMC or PFOR pathways, either in combination or independently, for acetate assimilation. In contrast, genomic analyses of *Rp. palustris*, another *icl* ^+^ organism, revealed absence of the EMC pathway while PFOR activity was detected in *Rp. palustris* (Petushkova et al., 2021; Petushkova et al., 2019). Despite these insights, significant gaps remain, and further investigation is needed to fully elucidate their roles in acetate metabolism.

Propionyl-CoA is produced as an intermediate of both the BCAA degradation route and the EMC pathway or as the activated form of propionate (C_3_). Its subsequent conversion to TCA cycle intermediates occurs primarily via the methylmalonyl-CoA (MMC) pathway, leading to succinyl-CoA formation, or the methylcitrate pathway, producing OAA via the (Carter and Alber, 2015; Segura et al., 2021) (Figure 2). In *Rs. rubrum*, propionate is mainly assimilated through the MMC pathway which also corresponds to the last steps of the EMC pathway (Bayon-Vicente et al., 2020a; Segura et al., 2021). Other PNSB, including *Ce. sphaeroides* and *Rp. palustris*, have also been shown to possess the MMC pathway (Hädicke et al., 2011; Erb et al., 2007; Alber et al., 2006) and use it for propionate assimilation (Petushkova et al., 2019; Schneider et al., 2012b). In *Rh. capsulatus*, RNA-seq data from a study conducted on 1,2-propanediol assimilation revealed that MMC genes were expressed under anaerobic conditions. Therefore, it is likely that the MMC pathway also plays a role in the assimilation of propionyl-CoA (Schindel et al., 2019). Interestingly, when propionate and butyrate are available as a mixture, *Rs. rubrum* seems to rely more on the methylcitrate (Figure 2) pathway for propionate assimilation than in propionate only conditions (Segura et al., 2021) where it primarily relies on the MMC pathway (Segura et al., 2021). In contrast, *Ce. sphaeroides* does not appear to use the methylcitrate pathway, as genes for 2-methylcitrate synthase and 2-methylcitrate dehydratase have not been identified yet in its genome, according to KEGG database. Moreover, BLAST analysis of the genes encoding these two enzymes in *Rs. rubrum* did not reveal any significant matches in *Ce. sphaeroides* genome (Altschul et al., 1990). Meanwhile, the genome of *Rh. capsulatus* contains all necessary methylcitrate pathway genes for converting propionyl-CoA to succinyl-CoA; however, experimental validation is needed to confirm the functionality of this pathway under photoheterotrophic conditions (Petushkova et al., 2019). For *Rp. palustris*, methylcitrate pathway genes show strain-specific variability, with only certain strains (e.g., BisB18 and BisA53) possessing the complete set of genes (Petushkova et al., 2019). In this way, although both the MMC and methylcitrate pathways are found in PNSB, the dominant pathways for propionate assimilation remain underexplored in many PNSB species.

McKinlay and collaborators investigated metabolic fluxes in *Rp. palustris* grown on 13C-labeled butyrate and found that butyrate is mainly metabolized via the glyoxylate shunt, with degradation through β-oxidation generating two molecules of acetyl-CoA. These acetyl-CoA molecules then enter the glyoxylate shunt, with only a minor flux passing through the PFOR pathway, converting acetyl-CoA to PA (McKinlay and Harwood, 2011). This suggests that the pathways supporting acetate assimilation in *Rp. palustris* are likely employed during butyrate metabolism as well. In *Rs. rubrum*, a study conducted by De Meur and collaborators (De Meur et al., 2020) demonstrated that butyrate can be converted into crotonyl-CoA (C_4_) by the butyryl-CoA dehydrogenase, allowing it to enter the EMC pathway (Figure 2). Interestingly, crotonyl-CoA carboxylase/reductase (CCR; EC: 1.3.1.85), one of the key enzymes of the EMC pathway, was found to be non-essential for growth under these conditions, unlike the other enzymes of the EMC pathway, as demonstrated by RB-TnSeq analysis. This finding suggests the potential for an alternative route from butyryl-CoA (C_4_) to ethylmalonyl-CoA that bypasses CCR. Supporting this hypothesis, Olsen and Merrick showed that propionyl-CoA carboxylase (EC: 6.4.1.3) could, at a lower rate, catalyze butyryl-CoA carboxylation to ethylmalonyl-CoA (Olsen and Merrick, 1968). This additional role of propionyl-CoA carboxylase may explain why the EMC pathway is required for butyrate assimilation, even though CCR is not. Alongside the EMC pathway, the isoleucine biosynthesis and degradation pathway, also named the methylbutanoyl-CoA (MBC) pathway (Figure 2), has also been demonstrated as an important pathway for *Rs. rubrum* growth in the presence of butyrate (De Meur et al., 2020). Nevertheless, the EMC pathway appears to be the preferred route in *Rs. rubrum* when butyrate is the only carbon source (De Meur et al., 2020). Despite butyrate’s role in supporting photoheterotrophic growth of PNSB species, the details on its assimilation pathway(s) in *Rh. capsulatus* are not well-defined (Richardson et al., 1988; Cabecas Segura et al., 2022a). It can be hypothesized, based on mechanisms observed in other strains, that *Rh. capsulatus* may rely on β-oxidation to initially process butyrate into acetyl-CoA, which could then enter the glyoxylate shunt, similarly to *Rp. palustris*, another *icl* ^*+*^ species (McKinlay and Harwood, 2011). Recent studies offer insights into the behavior of three PNSB species—*Rs. rubrum*, *Rh. capsulatus*, and *Ce. sphaeroides*—in assimilating synthetic VFA mixtures composed of acetate, propionate, and butyrate (Segura et al., 2021; Cabecas Segura et al., 2022a).

Besides these well-described assimilation pathways, the assimilation routes for longer-chain VFAs, specifically valerate (C_5_) and hexanoate (C_6_) are poorly documented in PNSB. In *R*s *rubrum*, recent findings indicate that valerate assimilation under photoheterotrophic conditions may begin with β-oxidation, yielding acetyl-CoA and propionyl-CoA (Figure 2). Acetyl-CoA can enter several metabolic pathways, such as the EMC pathway, the PFOR pathway, and the BCAA biosynthesis pathway, but only the latter two seems to be important for valerate assimilation as multiple enzymes of the BCAA biosynthesis pathway were found to be required for *Rs. rubrum* optimal growth on valerate (Bayon-Vicente et al., 2020a). The MMC pathway appeared to be crucial for valerate derived propionyl-CoA assimilation.

Hexanoate assimilation is *Rs. rubrum* follows a similar β-oxidation sequence, yielding one molecule of acetyl-CoA and one of butyryl-CoA (Cabecas Segura et al., 2022b) (Figure 2). These intermediates are subsequently integrated into anaplerotic pathways, such as the EMC and MBC pathways, both of which produce propionyl-CoA, which is then likely assimilated through the MMC pathway.

While research is less extensive in other PNSB strains, it has been shown that *Rh. capsulatus* (strains B10 and SB1003) can use longer-chain VFAs, including valerate and hexanoate, for both growth and polyhydroxyalkanoate (PHA) production under phototrophic conditions with bicarbonate supplementation. However, growth tests on *Rh. capsulatus* B10 indicated that its growth rate decreases with increasing fatty acid chain length (Kranz et al., 1997). This capability of assimilating longer-chain VFAs, particularly valerate, was shown to be shared *Rp. palustris* and *Ce. sphaeroides* (Liebergesell et al., 1991). Furthermore, in *Ce. sphaeroides*, a primary fatty acid kinase (RsAck; acetate kinase; EC: 2.7.2.1) offers an alternative route for VFA activation by phosphorylating VFAs, facilitating their entry into central metabolism (Bachochin et al., 2021). Despite these advances, the exact pathways for valerate and hexanoate assimilation in PNSB remain partially understood only, in *Rp. palustris*, *Rh. capsulatus*, and *Ce. sphaeroides*, necessitating further research to clarify these metabolic routes. An overview of the characterization status of the metabolic pathways mentioned above in the four PNSB strains described is provided in Table 1.

**TABLE 1:** Overview of the different metabolic routes involved in VFA metabolism under photoheterotrophic conditions and their characterization state in *Ce. sphaeroides*, *Rh. capsulatus*, *Rs. rubrum* and *Rp. palustris*.

Metabolic pathway	PNSB species			
	*Ce. sphaeroides* (*icl* ^*−*^)	*Rh. capsulatus* (*icl* ^+^)	*Rs. rubrum* (*icl* ^*−*^)	*Rp. palustris* (*icl* ^+^)
TCA cycle	+	+	+	+
Glyoxylate shunt	−	+	−	+
Ethylmalonyl-CoA pathway	+	+	+	?
Methylmalonyl-CoA pathway	+	+	+	+
Methylcitrate pathway	?	+ (theoretically; only based on gene presence)	+	+ Strain specific
Pyruvate:ferredoxin oxidoreductase (PFOR)	?	+	+	+
Methylbutanoyl-CoA pathway	+	+	+	+

Symbols indicate: (+) if the pathway has been described as present in the strain; (−) if the pathway has been described as absent in the strain; and (?) if genes of the pathway have not been identified yet in the strain. Icl^−^, isocitrate lyase-negative species; icl^+^, isocitrate lyase positive species.

Fermentation processes always lead to the production of mixture of VFA, however only few studies analyzed the metabolic response of PNSB exposed to such mixture. When provided with a mixture of propionate and butyrate, *Rs. rubrum* appears to still rely on the EMC pathway, but with an increasing implication of the MBC pathway (Segura et al., 2021). Moreover, when provided with a mixture of acetate, propionate and butyrate, *Rs. rubrum* and *Ce. sphaeroides*, displayed a sequential assimilation, with butyrate being consumed after acetate and/or propionate levels decreased. In contrast, *Rh. capsulatus* simultaneously assimilated all VFAs (Cabecas Segura et al., 2022a). These results therefore suggest that if assimilation pathways for VFA start to be well characterized (at least for the shorter chain VFA), mor research will be needed to better understand how PNSB assimilate VFA when provided in a mixture, a situation much closer to what is expected in AFW valorization.

Besides replenishing TCA cycle intermediates, most of the aforementioned pathways also play a role in maintaining redox balance in PNSB. Under photo-organo-heterotrophic conditions, PNSB face redox stress when assimilating highly reduced compounds such as VFAs (Table 2) which can be further reinforced by increased NADH production via the reverse action of NADH dehydrogenase, under sudden light intensity increases (hereafter referred as light stress) (Bayon-Vicente et al., 2020b; Herter et al., 1998; McEwan, 1994; Golomysova et al., 2010). To cope with this excess of reducing power, PNSB primarily rely on two mechanisms: CO_2_ fixation through the Calvin-Benson-Bassham (CBB) cycle (Gordon and McKinlay, 2014; McKinlay and Harwood, 2010) (or reductive pentose phosphate cycle) and H_2_ production, notably via nitrogenases (McKinlay and Harwood, 2011; McKinlay and Harwood, 2010; McCully and McKinlay, 2016; Farmer et al., 2014). The CBB cycle has been widely recognized in PNSB as the main route for redox balancing (McKinlay and Harwood, 2010; Wang D. et al., 2010; Falcone and Tabita, 1991), consuming two NADPH molecules for each CO_2_ molecule fixed. In this way, PNSB mutants lacking key CBB enzymes exhibit impaired growth on reduced substrates like malate (Gordon and McKinlay, 2014; Falcone and Tabita, 1991; Öztürk et al., 2012), succinate (Gordon and McKinlay, 2014; Hallenbeck et al., 1990), and acetate (McKinlay and Harwood, 2010; Laguna et al., 2011) underscoring the importance of the CBB cycle in managing the excess reducing power. Moreover, CO_2_ proved to be essential for the photoheterotrophic growth of PNSB on various VFAs such as butyrate (De Meur et al., 2020; McKinlay and Harwood, 2011; Ormerod, 1956), propionate (Ormerod, 1956), valerate (Bayon-Vicente et al., 2020a), and hexanoate (Cabecas Segura et al., 2022b). In some PNSB species like *Rs. rubrum* and *Ce. sphaeroides*, the EMC pathway was shown to reduce CBB requirement and allow light stress tolerance during acetate assimilation (Bayon-Vicente et al., 2020b; Laguna et al., 2011).

**TABLE 2:** Theoretical electron content of various VFAs compared to the theoretical electron content of PNSB biomass.

Compound	Formula	Electron content (mol e^−^. mol C^−1^)
Acetate	C_2_H_4_O_2_	4.0
PNSB biomass	CH_1.7_O_0.4_N_0.2_P_0.01_	4.3
Propionate	C_3_H_6_O_2_	4.7
Butyrate	C_4_H_8_O_2_	5.0
Valerate	C_5_H_10_O_2_	5.2
Hexanoate	C_6_H_12_O_2_	5.6

The elemental composition of *Rs. rubrum* biomass was determined under photoheterotrophic conditions in a previous study by Favier-Teodorescu et al. (2003). The theoretical electron content, expressed in moles of electrons per mole of carbon, was calculated based on the oxidized form of CO_2_, following the method previously applied by Alloul et al. (2023).

In addition to CO_2_ fixation and H_2_ production, many additional metabolic pathways have been proposed to participate in redox homeostasis such as polyhydroxyalkanoate (PHA) (Bayon-Vicente et al., 2020a; Leroy et al., 2015; Bayon-Vicente et al., 2020b; Hauf et al., 2013), isoleucine production (De Meur et al., 2020; Bayon-Vicente et al., 2020b; Bayon-Vicente et al., 2021; McCully et al., 2020), or the reverse TCA cycle (McCully et al., 2020; Joshi and Tabita, 2000). However, the reverse TCA cycle is not available in *Rp.* Palustris which lack fumarate reductase (McCully et al., 2020). PFOR activity also represents a key point in acetate metabolism, potentially contributing to redox balance (Leroy et al., 2015; Bayon-Vicente et al., 2020b) by facilitating acetyl-CoA carboxylation to pyruvate in anaerobic conditions (St. Maurice et al., 2007) (Figure 2). Additionally, PFOR channels acetyl-CoA into isoleucine biosynthesis, a putative electron sink especially in *Rs. rubrum* under acetate (Leroy et al., 2015; Bayon-Vicente et al., 2020b), butyrate (De Meur et al., 2020), valerate (Bayon-Vicente et al., 2020a) and/or light-stress conditions (Bayon-Vicente et al., 2020b). Taken together, these findings illustrate the highly flexible nature of redox-balancing strategies in PNSB, depending on various factors such as carbon substrate, CO_2_ availability, and light intensity.

### 3.2 AFW conversion to volatile fatty acids

AFW are an ideal substrate for VFA production through acidogenic fermentation (Jiang et al., 2013) due to their high biodegradability, high moisture content (Selvam et al., 2021; Zhang et al., 2007; Zhou et al., 2018) and organic matter composition. Anaerobic digestion (AD) is a sequence of microbial reactions taking place under anaerobic conditions and gradually degrading complex organic materials usually to produce biogas (Dong et al., 2023). AD occurs in four stages: i) hydrolysis, where complex organic polymers (e.g., polysaccharides, proteins, lipids) are broken down into simpler soluble monomers; ii) acidogenesis during which these intermediates are fermented by acidogenic bacteria into VFAs (mainly acetic, propionic, and butyric acids) (Zhou et al., 2018; Gould and Dahiya, 2015; Marquez et al., 2014; Sarkar et al., 2018). Amino acid degradation also produces ammonia gas (NH_3_) (Kamusoko et al., 2022). The subsequent stages—iii) acetogenesis, which converts H_2_ and CO_2_ into acetate, and iv) methanogenesis, which produces biogas (i.e., CH_4_ and CO_2_) (Zhou et al., 2018; Sarkar et al., 2018) are bypassed in dark fermentation, which halts the process after acidogenesis, focusing solely on VFAs and H_2_ production (Aghapour et al., 2020; Chong et al., 2009). The most abundant VFAs produced through anaerobic digestion include acetate, propionate, iso-butyrate, n-butyrate, iso-valerate, and n-valerate (Dahiya et al., 2018; Jiang et al., 2013; Aghapour et al., 2020; Sukphun et al., 2021), all of which can be efficiently assimilated by PNSB, as described hereabove. Producing hexanoate, a longer-chain VFA, however, requires an additional step known as chain elongation where shorter-chain VFAs such as acetic acid undergo reverse β-oxidation cycles, typically fueled by electron donors such as ethanol. As a result, hexanoate, though valuable, is less prevalent among directly produced VFAs in anaerobic digesters (Dahiya et al., 2018; Dong et al., 2023; Cavalcante et al., 2017). To enhance VFA production from AFW, various pretreatment methods, such as thermal, chemical, or microwave treatments, are applied (Strazzera et al., 2018). The fermentation process, and thus the VFA composition, is influenced by several key operational factors, including pH, temperature, hydraulic retention time (HRT) (i.e., the time the substrate and biomass remain in the reactor) and organic loading rate (OLR) (Mohan et al., 2016).

As previously mentioned, VFAs have a wide range of industrial applications, especially as key feedstocks in the production of biofuels and bioplastics (Merrylin et al., 2020). However, if VFA are produced from food waste through anaerobic digestion, food-grade materials are used for non-food-grade applications, which raises ethical concerns about diverting edible resources to industrial processes. This situation presents an opportunity to utilize VFAs as renewable carbon sources for the production of PNSB biomass. Many AFW could be converted to VFA through AD. Gottardo and collaborators (Gottardo et al., 2022) recently reviewed VFA production from cheese whey and winery wastewater. Spent mushroom compost (SMC) is also gaining interest, even though produced amounts are limited (8 million tonnes per year in China) (Fang et al., 2016). Highest VFA concentration achieved (Fang et al., 2016) was 2,781 mg/L with this substrate, acetic and propionic acids constituting the main components (collectively 71%) of the generated VFAs. Interestingly the composition of produced VFA seems to be particularly stable, thanks to stable composition of SMC as compared with other organic waste, which represent an asset for PNSB production.

Rice husk is another abundant AFW representing over 113 million tonnes (Yu et al., 2009), the main part of which are produced in China and India (Baetge and Kaltschmitt, 2018). To enhance VFAs production from rice husk, various pretreatment methods, including ultrasonic, thermal, acid, ozone, combined, and alkaline treatments, have been explored. Among them alkaline treatment is particularly effective in suppressing methanogens and promoting VFA accumulation up to around 1,200 mg·L^−1^ of VFA. Acetic acid and propionic acid were the main VFAs produced.

Potatoes are the main vegetables consumed by humans worldwide. The potato peel wastes contain starch and proteins which are ideal fermentation substrates. In 2023, Lu et al., determined that the VFA production issued from potato wastes fermentation reaches up to 22 gCOD/L, with butyric acid and acetic acids (∼12 gCOD/L and 8 gCOD/L, respectively) as most abundant VFAs (Lu et al., 2023). The anaerobic digestion of cucumber, tomato and lettuce into VFA has also been studied in batch and continuous mode reactors (Strazzera et al., 2018; Greses et al., 2020; Magdalena et al., 2019). The VFA profile was similar in all 3 waste types, with butyric and acetic acids being the most abundant. However, differences in the concentration of VFA could be observed, with bioconversion efficiencies of 58.0, 49.5% and 55.2%, respectively.

Aside from this specific industrial streams, Food waste (FW) from households, canteens or supermarkets represent a major feedstock for anaerobic digestion in terms of abundance reaching up to 40% of total food losses in industrialized countries (Stenmarck et al., 2016; Gustavsson et al., 2011; Strazzera et al., 2018). The nature of food waste is crucial to the efficacy of the process and the chemical composition of the resulting VFA. Moreover, the solid retention time and pH of an anaerobic digestor containing household waste was shown to impact the length of the produced VFAs with short chain VFAs (e.g., acetic and propionic acids) dominating during the early stage (up to 15 days), whereas longer chain VFAs accumulate after 15 days (Khatami et al., 2021). While the abundance of such AFW should encourage the development of PNSB-based process to valorize their VFA, the variability of their content could represent a huge challenge. In a study by Allegue et al. (2020), restaurant food waste was pre-treated thermally and anaerobically digested. The digestate was then use for the production of protein-rich PNSB, (67% of protein on dry weight basis) (Allegue et al., 2020).

### 3.3 Challenges and opportunities

So far, most studies on PNSB metabolism have focused on single carbon sources, yet VFAs in anaerobic fermentation effluents are typically found in complex mixtures, including acetate, propionate, (iso/n)-butyrate, and (iso/n)-valerate, as previously mentioned. VFA metabolism in PNSB varies considerably, not only between single and mixed VFA conditions but also depending on the strain. VFA assimilation pattern was also demonstrated to be strain-dependent, with *icl* ^*-*^ strains as *Rs. rubrum* and *Ce. sphaeroides* assimilating VFAs sequentially, with acetate and/or propionate inhibiting butyrate assimilation, while *icl* ^*+*^ strains such as *Rh. capsulatus* can assimilate multiple VFAs simultaneously. In this way, despite advances in understanding VFA assimilation in PNSB, the metabolic behaviour of the different strains under mixed VFA conditions remain poorly understood, complicating predictions of bacterial growth and biomass composition. Further research is therefore crucial for optimizing PNSB-based biotechnologies, enabling more efficient use of VFA mixtures without compromising overall process efficiency.

As previously mentioned in the sugar section, one of the main challenges of using agri-food waste for food or feed applications using PNSB biomass is maintaining an axenic process. Unlike sugars, which are high-energy compounds and can be readily fermented by a wide range of microbes under anaerobic conditions, VFAs are end products of fermentation. They represent a low-energy state, meaning they cannot be further fermented, which restricts their assimilation to a narrower group of bacteria. This specificity minimizes contamination risks in microbial cultures using VFAs as substrates. Under anaerobic conditions, PNSB exhibit a unique competitive advantage due to their versatile photoheterotrophic metabolism, being able to assimilate a wide range of organic substrates (i.e., organic acids, amino acids, alcohols, and sugars) under light exposure with no oxygen requirements, while efficiently converting carbon sources into biomass with conversion yields near 100% (Trüper et al., 1981; Madigan et al., 2009). This selectivity can make VFA-based processes inherently more resilient to contamination, allowing for more stable microbial cultures and reducing the need for strict sterilization protocols. Consequently, VFA-based systems can be advantageous in industrial applications where maintaining pure cultures is challenging, supporting more sustainable and robust production processes.

Apart from metabolic challenges and opportunities, using VFAs from acidogenic fermentation of AFW as carbon substrates comes with several operational challenges.

Even though a wide range of organic-rich wastes can serve as a substrate for acidogenic fermentation, as described hereabove, certain waste types, particularly solid and complex substrates like lignocellulosic biomass, require pretreatment to improve their biodegradability and thus VFA production. Indeed, the hydrolysis rate can be hindered by lignocellulosic compounds, fats, and proteins in food waste, which slow down microbial digestion. Pretreatment methods, whether physical [e.g., (hydro)thermal, ultrasound or microwave treatments], chemical, or biological, increase substrate solubilization, thereby enhancing the efficiency of hydrolysis, the latter being the rate-determining step in anaerobic digester (Fdez.-Güelfo et al., 2011; Park et al., 2005; Owusu-Agyeman et al., 2021; Lee et al., 2014). Physical treatments such as ultrasound and microwaves are effective but often have limitations due to high energy demands and costs. On the other hand, chemical pretreatments, including acid, alkali, ozone, and hydrogen peroxide treatments, may introduce toxic compounds that could inhibit certain microbial activities. Biological pretreatments using hydrolytic enzymes, specific bacterial strains, or fungal species also facilitate hydrolysis without generating toxic by-products and being too energy-intensive, though they can be costly and generally slower than chemical or physical methods [reviewed by Ramos-Suarez et al. (2021)]. In addition to pretreatment type, optimizing various digester parameters, such as pH, temperature, HRT, OLR, substrate composition, inoculum type, redox conditions and reactor operating conditions, is crucial for enhancing VFA production during acidogenic fermentation (Dahiya et al., 2018; Lee et al., 2014; Ramos-Suarez et al., 2021; Cremonez et al., 2021). These factors significantly impact VFA distribution and fermentation efficiency, leading to considerable variability across processes. For instance, the composition of food waste plays a critical role in determining the type of VFAs produced; the degradation of amino acids or the acidification of long-chain fatty acids primarily yields acetic acid, while the acidification of monosaccharides can produce a mixture of acetic, propionic, and butyric acids (Dahiya et al., 2018). To maximize VFA accumulation, various strategies that boost hydrolysis rates and promote acidogenesis should be employed, alongside deeper investigation into acidogenesis parameters such as operating pH to refine the overall process (Wang et al., 2014). Indeed, among the digester parameters, pH is one of the most critical, affecting both VFA yield and distribution [particularly acetic, propionic and butyric acids production (Bengtsson et al., 2008; Horiuchi et al., 1999; Yu and Fang, 2003; Horiuchi et al., 2002)], and the activity of the different microbes involved in acidogenic fermentation (Sukphun et al., 2021). Inhibiting methanogenic activity also offers key advantages, supporting VFA production efficiency and economic viability. Unlike methanogens, which have a slow growth rate (De Groof et al., 2021) and require a long HRT of 14–40 days (Srisowmeya et al., 2020; Calt, 2015) to produce biogas, acidogenic bacteria can achieve maximum VFA yield within 2–4 days (Srisowmeya et al., 2020; Calt, 2015) (depending on cultivation mode), extending up to 15 days or more with complex feedstocks (Lee et al., 2014; Bolaji and Dionisi, 2017; Jankowska et al., 2015). Reducing HRT can therefore favor the rapid growth of acidogenic microbes and enhance VFA production by inhibiting VFA-consuming methanogens; it also reduces the need for larger reactors, thereby lowering both capital and operational costs (Sukphun et al., 2021; Worwag et al., 2019). Although the fermentation broth would require centrifugation and microfiltration before being provided to PNSB, this approach could remain more cost-effective than VFAs isolation techniques required for chemical valorisation of these compounds [reviewed in Ramos-Suarez et al. (2021)]. By providing VFAs as a carbon source, PNSB can convert food-grade organic waste into biomass suitable for food-grade applications, preserving the material within the food production chain and enhancing the value of agri-food waste-derived products. Digestate used as a substrate for PNSB cultivation would also provide, in addition to VFA as carbon source, the necessary nitrogen and phosphorus, in the form of ammonium and phosphates (Drosg et al., 2015). Indeed, in AD, the degradation of proteins primarily converts organic nitrogen into ammonium nitrogen (Makádi et al., 2012). However, the high ammonium levels (1,500–7,200 mg/kg dry matter (Opatokun et al., 2016; Manu et al., 2021)) also pose a challenge, as excessive concentrations could hinder PNSB growth or even become toxic (Manu et al., 2021) making nutrient adjustments, such as dilution, necessary. Moreover, the production of VFAs during acidogenic fermentation contributes to a significant decrease in pH in the fermentation broth. Since PNSB generally thrive at a pH range between 6.5 and 7.5 (Imhoff et al., 2005), the acidic nature of digestate may necessitate the addition of buffering solutions to maintain an optimal growth environment.

## 4 CO_2_ metabolism in purple non-sulfur bacteria, opportunities and challenges for agri-food CO_2_-containing streams upcycling

The treatment of organic waste using AD does not only produce VFAs, but it mainly intends to produce biogas. The previous section concentrated on the upcycling of VFA that could be obtained through AFW anaerobic digestion. The following section will discuss the upcycling of CO_2_-containing streams. As the biogas contains methane (CH_4_), carbon dioxide (CO_2_) and low quantities of contaminating gases, PNSB could be used to upgrade the biogas by capturing CO_2_. CO_2_ is also produced during other fermentative processes, such as alcoholic beverage production. Capturing this CO_2_ would decrease the climate impact of the considered process but also represent a fantastic upcycling potential if food-grade CO_2_, today entirely released to atmosphere could be converted into food or feed.

### 4.1 CO_2_ metabolism in purple non-sulfur bacteria

PNSB are capable of carbon capture and utilization (CCU) thanks to the high metabolic versatility surrounding their CBB cycle. Even if CO_2_ assimilation is often dedicated to the mitigation of the redox stress (Alloul et al., 2023), PNSB are also able to use CO_2_ as sole source of carbon thus performing photoautotrophy (Madigan et al., 2009). Under photoautotrophy, cells assimilate CO_2_ via the carboxylation of ribulose-1,5-bisphosphate (RuBP), by the RuBP carboxylase/oxygenase (RuBisCO). Multiple subsequent reactions yield to the production f glyceraldehyde-3-phospate G3P which contribute to biosynthesis. The CBB assimilates 1 mol of CO_2_ (in the form of G3P) at the cost of 3 mol of ATPs and 2 mol of NADPH (Gordon and McKinlay, 2014; Wang et al., 2011). Even if the CBB cycle constitutes the main CO_2_ assimilation pathway, other central carbon metabolism pathways are known to allow the assimilation of carbon dioxide. Among them, the already mentioned EMC, MMC or PFOR pathways and the reverse TCA cycle are the most widely studied (Bayon-Vicente et al., 2020a; Leroy et al., 2015; Bayon-Vicente et al., 2020b; Williams et al., 2006) (Figure 2). Moreover, as already mentioned above, CO_2_ fixation can also serve as electron sink under photo-organo-heterotrophy in the presence of highly reduced carbon sources such as VFA (McKinlay and Harwood, 2010).

### 4.2 CO_2_-containing agri-food streams

#### 4.2.1 Biogas

Biogas production through anaerobic digestion is currently a well-developed approach to produce biobased and renewable fuels. Indeed, biogas utilization not only reduces GHG emissions but also enhances the circular bioeconomy by improving biowaste management (Rosen and Ödlund, 2019). In Europe, biogas use grew more than fivefold between 2005 and 2017. Moreover, the number of biogas facilities increased significantly, from approximately 10,500 in 2010 to about 19,000 in 2020 (Pavicic et al., 2022), demonstrating a clear drive toward establishing a more circular bioeconomy.

Biogas is a mixture of CO_2_ (up to 50%) and methane (up to 75%). To be more easily exploited as an energy source, biogas must be converted to biomethane to reduce contaminants and increase the energy yield from its combustion (Mekonen et al., 2023). Various physical and chemical transformation strategies are currently employed to separate CO_2_ and CH_4_ (i.e., membrane separation, water scrubbing, …) (Pavicic et al., 2022). In the context of a circular bioeconomy, alternative strategies using microorganisms are currently being explored. Biological transformation of biogas could be achieved using microalgae or cyanobacteria, with performance being better than other physico-chemical biogas-upgrading processes (Ferella et al., 2019; Toledo-Cervantes et al., 2017). However, the main drawback of this method is the production of oxygen, which must be stripped from the resulting biomethane (Marín et al., 2019). PNSB could therefore be interesting candidates for biogas refining thanks to their ability to grow in the presence of CO_2_. However, studies using PPB for biogas upgrading are sparse. A study by Marín et al. (2019) demonstrated that PPB could transform biogas into 93.3% CH_4_ by fixing CO_2_ and simultaneously utilize the VFA present in piggery wastewater (Marín et al., 2019). In this study, researchers compared the capacity of a biogas-upgrading system using algal-bacterial consortium to a PPB-based system. The biogas-upgrading system based on PPB showed a better reduction in the CO_2_ concentration of the biogas compared to the algal-bacterial consortium system. This was reflected by a decrease in CO_2_ headspace concentration from 28.6% to 3.3% in the PPB-based system and from 28.6% to 24.1% in the algal-bacterial consortium system. It is essential to mention that the use of any non-food by-product for biogas or VFA production would exclude the produced PNSB for feed or food application.

#### 4.2.2 Gases from fermentation processes

Other sources of CO_2_ could be explored as well, such as exhaust gases from fermentation in the context of alcoholic beverage production. Brewery and wineries are for example producing large amount of biogenic, contaminant free CO2-rich gas, which is most of the time not reused especially in smaller production plant. Such streams were already demonstrate to be compatible with Microbial ElectroSynthesis for the production of acetate (Roy et al., 2021). The direct emission of CO2 beer production theoretically reach 4 kg per produced hectoliter (at 4% alcohol content v/v) (Grand et al., 2024). In wine production, this level could raise by a factor of three. This biogenic CO2 is attracting more and more attention for beverage carbonation but could also be used for food-grade PNSB biomass production. A study using pure cultures of *Rh. capsulatus*, *Ce. sphaeroides* and *Rp. palustris* under photohydrogenotrophy (using electrons from H_2_ to fix CO_2_) demonstrated their ability to produce microbial protein at 2.6–2.9 g protein g^-1^ H_2_. These values were higher than those achieved by aerobic hydrogen-oxidizing bacteria, while their productivity exceeded that of photoautotrophic microalgae (Spanoghe et al., 2021). Moreover, bio-electrochemical processes using PNSB in photoautotrophy demonstrated their ability to fix CO_2_ using electrons from a biocathode (Vasiliadou et al., 2018). In these processes, the cathode serves as an electron donor, although other electron donors, such as ferrous iron have been described as well. In these mixed cultures, *Rhodopseudomonas* sp. played a crucial role in accepting extracellular electrons (Manchon et al., 2023a). Proof of concept using brewery wastewater containing 40 mM of acetate as a carbon and electron donor has been shown to outperform classical systems. These electrobiochemical systems represent sustainable alternatives for wastewater treatment, as they do not produce greenhouse gas emissions (Manchon et al., 2023b).

### 4.3 Challenges and opportunities

The efficiency of the anaerobic digestion process from food waste is dependent on various parameters (temperature, organic loading rate, etc.) as already mentioned in the above section, which might also influence the CO_2_ proportion in the biogas but also the presence of other gaseous component such as H_2_S. Interestingly, Egger et al. demonstrated recently that a natural consortium enriched in Purple Sulfur Bacteria (PSB) could be used for H_2_S removal from biogas (Egger et al., 2023). Some PNSB strains might also present H_2_S resistance phenotype. During the growth of PNSB in photoautotrophy, a source of electrons is required to fix the CO_2_. In the context of biogas upcycling, most research focuses on the use of cathodes, hydrogen or ferrous ions as electron donors (Spanoghe et al., 2021; Vasiliadou et al., 2018). However, using the VFAs remaining in the digestate to upcycle biogas could be interesting to investigate. Indeed, VFAs, being more reduced than PNSB biomass, their assimilation would generate a redox stress which would increase the CO_2_ fixation (Alloul et al., 2023). Such a process would have a two-fold advantage: eliminating the VFAs that could remain in the final digestate while upgrading the biogas to biomethane.

The major challenge of using PNSB to fix CO_2_ lies in ensuring adequate residence time for the gas within the liquid culture medium, as it must dissolve efficiently to be metabolized by PNSB. Enhancing gas dissolution requires maximizing the gas-liquid exchange surface. For instance, techniques such as “micro-sparging” (Nehring et al., 2004), which involves dispersing biogas into fine bubbles, combined with mixing or agitation, can significantly improve dissolution efficiency. However, these strategies come with trade-offs: they may reduce light penetration crucial for photosynthetic activity, introduce shear forces that could stress the microbial cells, and increase the complexity and cost of the equipment.

## 5 Alcohol metabolism in purple non-sulfur bacteria, opportunities and challenges for agri-food by-products upcycling

### 5.1 Alcohol metabolism in purple non-sulfur bacteria

Alcohols are organic compounds characterized by the presence of one or more hydroxyl groups attached to a carbon atom and answering the general formula C_n_H_n+2_OH (Henze and Blair, 1931).

The ability of PNSB to metabolize alcohols was first identified over 80 years ago (Foster, 1944; van Niel, 1944) and shows significant potential nowadays for enhanced processing of alcohol-containing waste streams. Specifically, alcohol dehydrogenases catalyze the oxidation of alcohols to aldehydes or ketones (Sojka et al., 1978), further oxidized to carboxylic acids by aldehyde/ketone dehydrogenases.

Research has predominantly focused on short-chain alcohols, which have been validated as suitable carbon sources for certain strains (Foster, 1944; van Niel, 1944; Sojka et al., 1978; Foster, 1940; Fujii et al., 1983; Quayle and Pfennig, 1975; Sahm et al., 1976; Siegel and Kamen, 1949; Yamanaka and Tsuyuki, 1983; Yamanaka and Minoshima, 1984) while longer-chain alcohols exhibit variable assimilation efficiency and remain understudied (Pantazopoulous and Madigan, 2000). Some alcohol dehydrogenases have been found to be sensitive to oxygen (Fujii et al., 1983; Sahm et al., 1976),suggesting a facultative anaerobic pathway for alcohol metabolism. Phototrophic growth conditions have been identified as the most efficient mode for cultivating PNSB on alcohol substrates, whereas chemotrophic conditions, though less extensively studied, resulted in limited to no growth (Pantazopoulous and Madigan, 2000). In *Rs. rubrum*, the alcohol dehydrogenase was shown to be inducible, with a 20-fold increase in activity compared to growth on malate, and NAD-dependent with NADP as a viable substitute (Sojka et al., 1978). However, these properties are not universal among PNSB. In *Rhodomicrobium vannielii*, the alcohol dehydrogenase is constitutive and strictly NADP-dependent (Sandhu and Carr, 1970), while in *Rhodopseudomonas acidophila* (*Rh. acidophila*), it was found to be inducible and flavonoid-linked (Yamanaka and Minoshima, 1984). To evaluate the potential of the four reference PNSB species for bioprocessing alcohol-containing waste, we assessed the presence of relevant enzymes within their genomes by consulting existing literature and, where needed, referencing pathways cataloged in the KEGG database Table 3. The primary alcohols under study include ethanol, methanol, and glycerol, as they are the most abundant in alcohol-rich waste streams. However, it is noteworthy that some PNSB can also process other types of alcohols, including short-chain (e.g., propanol, butanol, pentanol), long-chain (e.g., hexanol, though preculture induction is often required), aromatic (e.g., vanillyl alcohol), diols (e.g., 1,4-butanediol), and sugar alcohols (e.g., mannitol, sorbitol) (van Niel, 1944; Yamanaka and Tsuyuki, 1983; Pantazopoulous and Madigan, 2000). These processing capabilities show that the metabolic versatility of PNSB could also be used to process other alcohol-laden waste streams, provided that rigorous screening is undertaken to select the optimal strain.

**TABLE 3:** Growth of the four reference strains on three main alcohols found in waste streams according to literature and KEGG data.

Alcohols	*Rs*. *rubrum*		*Rh. capsulatus*		*Ce. sphaeroides*		*Rp. palustris*	
	Liter	KEGG	Liter	KEGG	Liter	KEGG	Liter	KEGG
Ethanol	+^a^	+_2_	−^a^ +^c^ −^d^ −^e^	+	+^a^ +^d^	+_4_	+^a^ ±^b^ +^d^	+_7_
Methanol	±^a^	−_2_	0^a^ −^e^	−	0^a^	+_4_	−^a^ −^b^	+_2_
Glycerol	−^a^	+_2_	−^a^ −^e^ ∼^f^	+	+^a^	+_4_	+^a^ ±^b^ +^g^ +^h^	+_7_

Symbols (liter.): +: growth, −: no growth, ±: growth for certain strains, ∼: mutants; 0: not studied yet. Sources: a: (Trüper et al., 1981); b: (Fujii et al., 1983); c: (Pantazopoulous and Madigan, 2000); d: (van Niel, 1944); e (Weaver et al., 1975); f: (Lueking et al., 1973); g: (Sabourin-Provost and Hallenbeck, 2009); h: (Pott et al., 2013). For the KEGG data, a symbol was added for each strain available. Symbols (KEGG): +_n_: at least one set of required enzymes present (in any metabolic pathway); −_n_: enzymes required not present; with “n” being the number of strains available on KEGG (Kanehisa and Goto, 2020).

Ethanol is first converted to acetaldehyde by an ethanol dehydrogenase, and subsequently to acetate via an aldehyde dehydrogenase (Fujii et al., 1983). Acetate can then be activated to acetyl-CoA and incorporated into cellular metabolism, as outlined in the VFA section. Given that ethanol is more reduced than PNSB biomass, its assimilation requires oxidation leading to redox imbalance and associated homeostasis reaction, as described earlier. Pioneering studies by Foster (Foster, 1944; Foster, 1940) explored the growth of 70 unclassified PNSB strains in the presence of various alcohols, where ethanol proved to be effective as enrichment for culture media at 0.2%. By 1944, Van Niel had documented the growth of *Rp. palustris* and *Ce. sphaeroïdes* on ethanol at 0.2% (van Niel, 1944), with subsequent confirmation in *Rs. rubrum*, also at 0.2% (Trüper et al., 1981; Fujii et al., 1983). Although earlier research reported no growth of *Rh. capsulatus* on ethanol (Trüper et al., 1981; van Niel, 1944; Weaver et al., 1975), more recent studies have observed limited growth (Pantazopoulous and Madigan, 2000) on ethanol at 0.2% (max 4%), supported by the genomic evidence of the presence of alcohol and aldehyde dehydrogenases in the KEGG database Table 3.

Methanol is generally converted to formaldehyde via a methanol dehydrogenase (Trüper et al., 1981), followed by a conversion to formic acid through a glutathione-dependent aldehyde dehydrogenase, and ultimately to CO_2_ via a hydrolase (Hickman et al., 2002; Jackson et al., 2009). While certain strains of *Rs. rubrum* (Trüper et al., 1981) and *Ce. sphaeroides* (Hickman et al., 2002; Barber and Donohue, 1998) can metabolize methanol, *Rp. palustris* (Trüper et al., 1981; Fujii et al., 1983) and *Rh. capsulatus* seem to lack that ability (Pantazopoulous and Madigan, 2000; Weaver et al., 1975). Other PNSB, such as *Rh. acidophila* 10050, can also use methanol in anaerobic conditions up to 125 mM (±0.4%), either as the sole carbon source or co-metabolized with another substrate (Quayle and Pfennig, 1975). The formaldehyde produced is then further oxidized into CO_2_ to be assimilated autotrophically by the CBB (Sahm et al., 1976). Just like ethanol, the assimilation of methanol will generate an excess of reducing power within the cell, justifying the need for an electron sinking mechanism such as CO_2_ assimilation via the CBB, hence the need for additional bicarbonates in the culture medium (Quayle and Pfennig, 1975).

Glycerol is usually phosphorylated to glycerol-3-phosphate by a glycerol kinase (EC 2.7.1.30), then dehydrogenated using a glycerol-3-phosphate dehydrogenase (EC: 1.1.1.94 or 1.1.5.3) further metabolized through the EMP or the pentose phosphate pathway (Sojka et al., 1978; Yamauchi et al., 2019; Lueking et al., 1973). The presence of these enzymes has been confirmed in *Rp. Palustris* and *Ce. Sphaeroides* at 100 mM (0.92%) of glycerol. Other studies have found that *Rhodospirillum rubrum* (Trüper et al., 1981) and *Rhodobacter capsulatus* (Weaver et al., 1975) lack the enzymes required for glycerol metabolism. However, spontaneous mutants of *Rh. capsulatus* have demonstrated the capacity for constitutive glycerol metabolism at 0.5% (54 mM) (Lueking et al., 1973; Spear and Sojka, 1984), and the KEGG database indicates the presence of these enzymes across multiple strains Table 3. This observation suggests that other factors may be inhibiting the utilization of glycerol, despite the genetic presence of the necessary enzymes.

### 5.2 Alcohol-rich agri-food by-products

From their use in various industrial processes and pharmaco-medical fields (e.g., solvents, antiseptics, disinfectants, personal care products) to their role as intermediates in the synthesis of chemical compounds (e.g., acetic acid, formaldehyde, ethylene, butyl acrylate), as well as their utilization as fuels and their role in the food industry (e.g., beverages, food preservation), alcohols are fundamental to sustain the quality of life in modern civilization (Kuhz et al., 2017). In the agrifood sector specifically, most alcohol-containing by-products are generated when carbohydrate fermentation is indissociable from the production of the compound of interest. For instance, breweries, wineries and distilleries generate many kinds of post-fermentation waste, such as stillage (e.g. pot ale, distillery slops, spent wash, vinasse), solid residuals (e.g. spent yeast, spent lees) or unusable and expired products (Conradie et al., 2015; Stewart et al., 2017; Stichlmair et al., 2021). However, these compounds generally present low alcohol concentrations, since alcohol-generating processes are optimized to avoid losses of the product of interest, which is the alcohol itself. In fact, the remaining high-alcohol fractions (e.g. low wines, feints) are often distilled again to minimize losses (Stichlmair et al., 2021).

Given that the specific alcohol content in waste streams of the fermentation industry largely depends on the process considered (Wilkie et al., 2000; Mosse et al., 2011), some case studies will be presented instead of approximations. In Mosse et al., winery wastewater presents alcohol levels as follows: 1–5 g.L^−1^ of ethanol, 0.14–0.32 g.L^−1^ of glycerol, 0–15 mg.L^−1^ of methanol, 0–5 mg.L^−1^ of 2-phenylethanol and i-amyl alcohol, 0–2 mg.L^−1^ of propanol, 0–1 mg.L^−1^ of n-butanol and 1-butanol (Mosse et al., 2011). In Conradie et al. the composition analysis indicates 4.9 g.L^−1^ of ethanol, 0.87 g.L^−1^ of glucose and fructose and 0.32 g.L^−1^ of glycerol (Conradie et al., 2015). Winery wastewater is usually pretreated (e.g., screening/settling, pH adjustment) before being processed using a variety of techniques, such as biological aerobic [e.g., aerated lagoons, activated sludge, and Sequencing Batch Reactors (SBR), etc] or anaerobic treatments (e.g.,; anaerobic SBR, upflow anaerobic sludge blanket, anaerobic lagoons, etc.) (Mosse et al., 2011). Given the high presence in winery waste of compounds like ethanol, methanol and glycerol streams PNSB could add significant value to anaerobic treatment processes by enhancing the breakdown of alcohol-rich waste while simultaneously processing the wide variety of high COD compounds present in most agri-food waste streams (Conradie et al., 2015; Mosse et al., 2011). In return, PNSB would also produce valuable products, notably edible biomass if the food grade status of the wastewater is maintained along the process.

### 5.3 Challenge and opportunities

The primary challenge in utilizing PNSB for alcohol-containing waste processing lies in the limited alcohol concentration tolerance these organisms have. In fact, most studies cultivate PNSB at around 0.2% of alcohol, which inherently restricts the rate at which waste streams can be processed. For instance, *Rh. capsulatus* B-10, when cultivated under phototrophic conditions, exhibits a maximum ethanol tolerance of 4% v/v, with optimal growth observed at just 0.2% v/v (Pantazopoulous and Madigan, 2000). Additionally, the growth rate on alcohol is quite low, especially considering that PNSB growth in photoheterotrophy can achieve growth rates up to 7.2 d^−1^. For instance, *Rh. capsulatus* can reach 2.70 d^−1^ under photohydrogenotrophic conditions (Spanoghe et al., 2021), compared to only 0.5 d^−1^ observed at 0.2% of ethanol (Pantazopoulous and Madigan, 2000). Furthermore, the culture conditions required for PNSB in alcohol-containing environments often differ substantially from their optimal growth media, complicating the screening and adaptation of strains for alcohol tolerance. Quayle and Pfennig, for example, demonstrated methanol tolerance in strains such as *Rhodopseudomonas gelatinosa*, *Rhodopseudomonas acidophila*, *Rhodospirillum tenue*, and *Rhodospirillum vannielii*; however, significant variations in culture conditions (e.g., pH, bicarbonate requirement, etc.) make it challenging to determine which PNSB strains could effectively metabolize alcohol as a carbon source (Quayle and Pfennig, 1975). Finally, the scarcity of recent research, with much of the relevant literature dating back approximately 40 years, ultimately constrains the development of alcohol-processing applications using PNSB, creating an impression of an underdeveloped field.

Despite these challenges, the use of PNSB for processing alcohol-containing waste streams offers several notable advantages. Due to the stringent culture requirements for PNSB in the presence of alcohol, the process is likely highly resistant to contamination, especially under anaerobic conditions, allowing the process to maintain food-grade quality standards. This is particularly valuable in the fermentative industry, where waste streams are generally considered “food-grade,” thus enabling the produced biomass to be used as potentially edible material for feed and food applications (Alloul et al., 2019; Alloul et al., 2021). Additionally, alcohol-containing waste often contains other valuable compounds, such as unfermented carbohydrates and proteins, which PNSB can readily metabolize thanks to their metabolic versatility.

## 6 Conclusion and perspectives

Even if the use of PNSB has already been investigated in the presence of several agri-food industry by-products, most of the research conducted focused on the valorization of these materials through the production of energy (i.e., biohydrogen) or bioplastics (i.e., polyhydroxyalkanoates). This valorization strategy constitutes a downcycling of the AFW leading to a loss of food-grade materials while sustainability requirement and food supply security would rather claim for the upcycling of AFW. The extraordinary metabolic versatility of PNSB combined with their unique carbon conversion yield turn these organisms into promising solutions for the upcycling of agri-food by-products. Indeed, considering their unique features (i.e., superior quality protein content, antioxidants, interesting lipid profiles, Q10 coenzyme, vitamins), PNSB represent promising organisms for food and feed applications. Moreover, today in force patents for aquatic animal feed composition and anti-cholesterol features further show the relevance of PNSB for the food and feed industry. However, despite the potential applications of the PNSB for AFW valorization, only a few studies were conducted on the understanding of PNSB metabolism in the presence of such by-products. Indeed, most of the fundamental research mentioned in this review has been conducted in the presence of synthetic medium composed of a restricted quantity of different carbon sources. Therefore, the comprehensive understanding of PNSB behavior in the presence of industrial and highly complex AFW is still missing. In the context of a metabolic knowledge-based optimization approaches, the elucidation of PNSB metabolism in the presence of AFW is required to enhance biomass productivity, strain selection or optimize redox balancing strategy. As an example, if considering a bioprocess containing sucrose as main carbon source, the use of *Rs. rubrum* as bacterial strain will impose the enzymatic pretreatment of sucrose to release fructose and glucose whereas the use of *Rh. capsulatus* would not. In that context, a complete understanding of the different PNSB metabolisms is mandatory to allow the highest valorization of the different AFW but also to grasp the full potential of a PNSB toward the largest range of AFW possible.

The AFW considered in this review are, for most of them, only produced seasonally or on a discontinuous basis which also represents some challenges for PNSB-based valorization. Indeed, it is unlikely that a PNSB production plant could be economically viable if operating only part time during the year. Consequently, the selected AFW will have to be stored, and its composition should thus be enough stable. Molasses stability is guaranteed by its high concentration preventing microbial development. However, a less concentrated sugar-rich stream would need immediate treatment. VFA containing digestate should be stable as this carbon substrate represent low energy content molecules but outgassing of these volatile elements should be prevent by proper containment. Alcohol and CO2 containing streams are usually stable by nature.

Another important challenge associated with valorization of all AFW is the composition variability. As mentioned, the composition of molasses, digestate or biogas is largely depending on the production process and primary feedstock quality. It is likely that, at least at early stage of industrialization, only larger production plant would produce reproducible AFW compatible with PNSB valorization. Variability in waste stream associated with smaller production scale would represent an additional huge challenge for the development of PNSB-based AFW valorization processes.

The use of AFW for PNSB cultivation is likely to be accompanied by one or multiple pre-treatment steps (i.e., mechanical, thermal or biological). Even if such pre-treatments represent common features in bioindustrial processes, they require optimization in order to yield adequate carbon composition for PNSB culture. Moreover, considering food and feed applications, the selection of the cultivation facilities represents a key parameter. Indeed, the upcycling of AFW, thus producing food/feed-grade biomass, implies that the whole process should be conducted under axenic conditions in order to ensure food chain security. Hence, the use of open photobioreactors (i.e., raceway ponds), while being cheaper, is inexpedient for food application and the PNSB cultivation is likely to rely on closed photobioreactors, even if this will be linked with higher operation costs. However, as observed with other phototrophic microorganisms, closed systems allows higher biomass productivity in PNSB. The trade-off between closed and open systems for phototrophic microorganisms cultivation has been extensively reviewed previously and is out of the scope of this manuscript (Capson-Tojo et al., 2020; Magalhães et al., 2022; Barboza-Rodríguez et al., 2024; Sukacová et al., 2021). Finally, even if many studies have been conducted on the optimization of culture parameters, a knowledge gap exists when considering downstream processing (i.e., harvesting, biomass stabilization). Consequently, already existing technologies are transferred to PNSB facilities yielding non-optimized solutions and thus lower productivity.

Finally, even if different studies paved the way for future food and feed applications, the use of PNSB is today limited by regulatory aspects (novel food or feed additive agreement required). EFSA and European commission regulation on novel food, and to a lower extend on feed additive, impose a strict identification of the candidate species. Even in this hypothesis of the approval of a coculture of bacteria for novel food, the composition (proportion of each strain) should be described as well and the marketed product would not be allowed to deviate significantly from this composition. While a coculture would undoubtedly bring tremendous benefits (higher robustness, larger metabolic capabilities…) to the development of PNSB-based AFW valorization, it is unlikely that it might be compliant with current European regulation. Novel food agreement today in force were all delivered for a specific organism (strain level) but also for specific cultivation, and downstream processes. It is also important to mention that attracting the intense investment needed to obtain a novel food agreement would probably require the associate protection of strain and production processes. Therefore, food and feed applications should most likely target single PNSB strain-based process instead of a community of organisms, further emphasizing the importance of the selection of the most adequate PNSB strain. Although this review points out some metabolic knowledge and technology gaps and challenges, PNSB-based processes represent one of the most encouraging alternatives to already existing AFW valorization processes. Moreover, thanks to their outstanding metabolic versatility PNSB constitute promising organisms to design the future photorefinery allowing to better valorize AFW.
